# Psychosocial well-being in young adults with chronic illness since childhood: the role of illness cognitions

**DOI:** 10.1186/1753-2000-8-12

**Published:** 2014-04-15

**Authors:** Eefje JA Verhoof, Heleen Maurice-Stam, Hugo SA Heymans, Andrea WM Evers, Martha A Grootenhuis

**Affiliations:** 1 Psychosocial Department, Emma Children’s Academic Medical Center, University of Amsterdam, Amsterdam, The Netherlands; 2 Department of Pediatrics, Emma Children’s Hospital, Academic Medical Center, University of Amsterdam, Amsterdam, The Netherlands; 3 Institute of Psychology, Health, Medical and Neuropsychology, Leiden University, Leiden, The Netherlands; 4 Psychosocial Department, Room A3-241, Emma Children’s Hospital, Academic Medical Center, PO Box 22660, 1100 DD Amsterdam, The Netherlands

**Keywords:** Illness cognitions, Young adults, Chronic illness, Health related quality of life, Anxiety and depression

## Abstract

**Background:**

More and more pediatric patients reach adulthood. Some of them are successfully integrating in adult life, but many others are not. Possibly Illness cognitions (IC) - the way people give meaning to their illness/disability – may play a role in individual differences on long-term adjustment. This study explored the association of IC with disease–characteristics and Health Related Quality of Life (HRQoL), anxiety and depression in young adults with a disability benefit due to childhood-onset chronic condition.

**Methods:**

In a cross-sectional study, young adults (22–31 years, N = 377) who claimed a disability benefit because of a somatic condition since childhood, completed the Illness Cognition Questionnaire (acceptance-helplessness-benefits), RAND-36 (HRQoL) and HADS (anxiety and depression) online. Besides descriptive statistics, linear regression analyses were conducted to predict (1) illness cognitions by age, gender and disease-characteristics, and (2) HRQoL (Mental and Physical Component Scale), Anxiety and Depression by illness cognitions, controlling for disease-characteristics, age and gender.

**Results:**

Respectively 90.2%, 83.8% and 53.3% of the young adults with a disability benefit experienced feelings of acceptance, benefits and helplessness. Several disease-characteristics were associated with IC. More acceptance and less helplessness were associated with better mental (β = 0.31; β = −0.32) and physical (β = 0.16; β = −0.15) HRQoL and with less anxiety (β = −0.27; β = 0.28) and depression (β = −0.29; β = 0.31).

**Conclusions:**

IC of young adult beneficiaries were associated with their HRQoL and feelings of anxiety and depression. Early recognition of psychological distress and negative IC might be a key to the identification of pediatric patients at risk for long-term dysfunction. Identification of maladaptive illness cognitions enables the development of psychosocial interventions to optimise their well-being and adaptation to society.

## Background

Due to improved treatment possibilities and the positive consequences for life expectancy, the number of chronically ill children who live for longer is increasing, and more pediatric patients with somatic conditions are living into adulthood [1,2]. For these children, transition into adulthood is a critical phase. Children and adolescents with chronic illnesses are expected to go through similar developmental stages as their healthy peers; they will leave home, develop psychosocially, and define their role in the community through employment or other activities [3]. For patients with impairments, reaching these developmental stages can be challenging. Research findings indicate that school-aged children with chronic conditions, regardless of their diagnosis, are more limited in their participation in everyday life than their peers [4,5]. Also, research has showed that adolescents and young adults with disabilities often follow atypical developmental patterns when compared to their peers without a disability [6-8] and that they are at risk of poor educational, vocational and social outcomes in adulthood [4,9-11].

In the Netherlands, some 500,000 children (14%) are growing up with a chronic condition and 90% of them will reach adulthood [1]. As a result, many patients with a childhood-onset chronic condition will reach the age at which they enter the labour market. In the Netherlands, young people who are partially or fully incapable of working, due to a childhood-onset chronic condition, may be eligible for a benefit under the scheme for young disabled persons: Wajong (the Invalidity Insurance Act for Young Disabled Persons). This Act provides income support as well as support to find employment and if necessary support at the work place. A Wajong benefit is payable no earlier than the 18th birthday, for as long as the inability to work lasts and ends when the recipient reaches the age of 65. The level of benefit received under the terms of the Act depends on age and the amount someone can earn from a job; Wajong income support is a supplementary payment on top of what a young person with a chronic disease or handicap is able to earn from work.

Although some of the young adults growing up with a childhood-onset chronic condition make a good adjustment to adult life, many others struggle with the impact of their condition on overall well-being and adaptation to social life [9,12,13]. The nature and magnitude of their problems in adult life can vary greatly from patient to patient, even in those with the same diagnosis. Symptom severity is often insufficient to fully explain their adverse effects upon functioning. The discrepancy between the level of illness-related dysfunction in the physical, mental and social domain and the underlying pathology of the disease has given rise to hypotheses about the contribution of psychosocial factors to health outcomes in patients with chronic illnesses [14]. When patients are diagnosed with an illness they generally develop an organised pattern of beliefs about their condition. These illness perceptions or cognitive representations directly influence the individual’s emotional response to the inherently aversive character of a chronic condition, to maintain a sense of balance and to achieve a satisfying quality of life. This in turn determines how patients respond to the chronic condition in their coping behaviour such as adherence to treatment [15,16]. It has been commonly assumed that the way adult patients perceive and think about their illness accounts for much of the individual differences in their physical and psychological health status [16]. Specifically, patients who report high levels of helplessness and low levels of acceptance with regard to their illness, emphasize the negative aspects of their condition. They generalize their illness cognitions, defined as cognitive reactions to an uncontrollable, long-term stressor of coping with and adjusting to a chronic condition [16], to all facets of daily life and consequently experience worse physical and psychological functioning [16-19]. In this view, illness cognitions (IC) can be considered as prognostic factors predicting physical functioning, psychological distress and therefore possibly adaption to society.

Many instruments that are used to assess illness cognitions measure them as disease-specific cognitions or as trait-like constructs unrelated to chronic illness [20]. Instruments that generalize across chronic diseases would offer an opportunity to study the common mechanisms that contribute to individual differences in health outcomes. Furthermore, most instruments focus on maladaptive cognitions that predict unfavourable long-term outcomes in chronic diseases. However, knowledge on both maladaptive and adaptive cognitions can contribute to fully understanding individual differences in adjusting to chronic illness [16]. It also can yield new opportunities for psychosocial support. Therefore, Evers et al. developed an instrument, the Illness Cognitions Questionnaire (ICQ), that assesses a basic set of three generic illness cognitions applicable across a range of chronic diseases and that focus on both unfavourable and favourable ways of adjusting to chronic diseases by emphasizing the negative meaning through helplessness cognitions, decreasing the negative meaning by acceptance cognitions or adding a positive meaning by cognitions of perceived benefits [16].

Most of the research on IC has focused on adults while much less attention has been paid to younger people who grew up with a chronic condition. Also, the IC of young adult beneficiaries as a group has never been studied. Since they can be considered as the most vulnerable young adults with chronic conditions - those who have to apply for disability benefits as a result of their conditions - it is important to know how and to what extent the IC affect their well-being. Given the increase in the number of children and adolescents with a childhood-onset chronic condition and the growing number of them applying for disability benefits, it is essential to gain insight into their IC in order to be able to develop strategies to support this vulnerable population towards adulthood independence. The present study focussed on generic IC in young adult beneficiaries (Wajong benefits as a result of a chronic somatic illness or disability since childhood) in order to be able to develop strategies for psychosocial support that might optimise well-being and adaptation in society. Specifically, this study examines (1) illness cognitions in young adults with Wajong benefits in relation to disease-characteristics, and (2) associations of illness cognitions with health related quality of life (HRQoL), anxiety and depression, independent of the contribution of disease-characteristics, age and gender. It was hypothesized that the illness cognitions acceptance and benefits are associated with a higher HRQoL and lower feelings of anxiety and depression and vice versa for helplessness.

## Methods

### Procedures

This study was conducted within the framework of a large cross-sectional study EMWAjong (a contraction of ‘EMMA’ (from Emma Children’s Hospital Academic Medical Centre) and ‘Wajong’ (the name of the disability benefit), a study directed at investigating psychosocial functioning in young adults with a Wajong benefit for a childhood-onset chronic somatic condition and the factors affecting their vocational success. There could be complex interactions between problems which are a direct result of the chronic condition and its treatment, and possible psychosocial problems, which are an underlying reflection of growing up with a chronic condition and effect on life and social/economic status of these young adults. This manuscript focuses on the type and magnitude of generic illness cognitions affecting the lives of young adults who have grown up with a somatic condition. In this article we will refer to this group as ‘young adults claiming disability benefits’. All young adults between 22 and 31 years of age who claimed a Wajong benefit in the year 2003 or 2004 for a chronic somatic condition were invited in 2009 to participate in EMWAjong via a letter. The invitation letter was printed on Emma Children’s Hospital paper and in the letter was clearly stated that the benefits agency wasn’t involved in this study. Participation meant completing an online questionnaire. Those with no sustainable work opportunities (classified as fully incapable for work) were excluded because the EMWAjong study aimed to identify factors that could help to improve vocational success. Those with serious cognitive impairment or psychiatric conditions were also excluded because the EMWAjong study was directed at young adults with childhood-onset somatic conditions. Besides, it may be assumed that other mechanisms play a role in growing up with psychiatric conditions than growing up with somatic conditions.

In total, 2,046 persons were invited to take part in the study. To maintain the privacy of the beneficiaries, the invitation letter was sent by UWV, the Dutch benefits agency. The letter contained a personal log in code, a password and a link to the online questionnaire. After two weeks, participants received a reminder letter. Participants who completed the entire questionnaire received a gift voucher. The study was performed according to the regulations of the medical ethical committee. Due to the once-only internet-based nature of the survey, no formal approval by the medical ethics committee was required.

### Participants

A total of 415 young adults with a chronic somatic condition participated in the study (response rate 20.1%). A total of 38 respondents were removed because of an incomplete ICQ; data from 377 participants could be used for the analyses: 243 women (64.5%) and 134 men (35.5%), mean age 25.0 years (SD = 2.1; range 22.5-30.9). Non-responders differed from responders with respect to gender; 51.4% vs. 64.5% women (p < 0.05). The study group consists of young adults with a variety of (consequences of) chronic conditions, e.g. childhood cancer, asthma, muscular diseases, blindness, trauma. The demographic and disease- characteristics of the EMWAjong group are listed in Table 1.

**Table 1:** **Characteristics of young adults with disability benefits**^**1**^

**Demographic characteristics**	**N**		**%**		
*Female gender*	243		64.5		
	*M*		SD	Range	
*Age at study (years)*	25.0		2.1	22.5 - 30.9	
**Disease characteristics**	**N**	**%**		**N**	**%**
					
*Congenital disorder*	200	53.1	*Daily medicine use*	196	52.0
*Perceptible disability*	159	42.2	*Medical aids/devices*	184	48.8
*Course of illness*			*Limitations in fingers/hand*	148	39.3
Worse/variable	156	41.4	*Limitation of sight*	90	23.9
Stable/positive	221	58.6	*Limitations of hearing*	31	8.2
			*Able to sit half an hour*	350	92.8
			*Able to stand half an hour*	220	58.4
**Chronic conditions**	**N**				
*Visually impaired/blind*	51				
*Spasm*	45				
*Rheumatoid arthritis*	45				
*Epilepsy*	31				
*Back complaints*	28				
*Hearing impaired/deaf*	27				
*Intestinal complaints*	21				
*CFS*	20				
*Lung complaints*	20				
*Accident damage*	19				
*Paralysis*	19				
*Cancer*	18				
*Muscular dystrophy*	17				
*Arthritis*	16				
*Kidney diseases*	13				
*Skin disease*	9				
*Migraine*	9				
*Heart disease*	7				
*Other*	119				

^**1**^ EMWAjong group; N = 377.

### Measures

#### Illness cognitions

Illness cognitions were assessed with the ICQ [16] that measures generic illness beliefs across chronic conditions. The ICQ is a 18-item questionnaire that contains three six-item scales related to cognitive ways patients ascribe meaning to chronic illness: helplessness (focusing on the negative consequences of the disease and generalizing them to functioning in daily life; e.g.: “My illness limits me in everything that is important to me”), acceptance (acknowledging being chronically ill and perceiving the ability to manage the negative consequences of the disease; e.g.: “I have learned to live with my illness”) and perceived benefits (also perceiving positive, long-term consequences of the disease, e.g.: “Dealing with my illness has made me a stronger person”). Items are scored on a four-point Likert scale (1 = not at all, 2 = somewhat, 3 = to a large extent, 4 = completely). Scale scores for the three illness cognitions are calculated by summing up the item scores. For each scale the mean item score was calculated by dividing the scale score by the number of the items, resulting in a mean item scale score from 1 to 4. Higher scores indicate that the illness cognition is stronger present in the respondent. The ICQ has strong internal consistency and reliability, and good construct and predictive validity across chronic conditions [16,20]. Cronbach's alpha in the present study was 0.84 for helplessness, 0.88 for acceptance, and 0.84 for perceived benefits.

#### Health related quality of life

HRQoL was assessed using the RAND-36. The RAND-36 is a Dutch version of the MOS-SF-36 Health Survey and is almost identical to the Dutch SF-36 [21]. The RAND-36 is a multidimensional questionnaire consisting of 36 items with standardized response choices, clustered in 8 multi-item scales: Physical Functioning (10 items), Social Functioning (2 items), Role limitations owing to Physical health problems (4 items), Role limitations owing to Emotional problems (3 items), general Mental Health (5 items), Vitality (4 items), Bodily Pain (2 items), and General Health perceptions (5 items). All raw scale scores were converted to a 0–100 scale, with higher scores indicating higher levels of functioning or well-being. The validity and reliability of the RAND scales were satisfactory [22]. Among the EMWAjong group we found Cronbach’s alphas of 0.75 to 0.95.

Overall physical and mental health was assessed by aggregating all scale scores according to the algorithm described by Ware and Kosinski [23], yielding to the so-called Physical Component Scale (PCS) and to the Mental Component Scale (MCS). The scale weights were derived from Principal Components Analysis (PCA) with the RAND-36 data of a Dutch reference group [24], using a non-orthogonal rotation (Oblimin), on the basis of the assumption that physical health and mental health are interdependent. According to the weights derived from the PCA, the eight scales of the RAND-36 are represented in the PCS and MCS. The PCS reflects the physical aspects of HRQoL; Physical Functioning, Role limitations owing to Physical health problems and Bodily Pain are most strongly represented. MCS reflects the mental aspects of HRQoL; general Mental Health, Role limitations owing to Emotional problems and Social Functioning are most strongly represented.

#### Anxiety and depression

Anxiety and depression were measured using the Hospital Anxiety and Depression Scale (HADS) [25]. The 14 items are scored on a four-point scale (0–3), producing a total score ranging from 0 to 21, for depression (7 items) – and anxiety (7-items). Higher scale scores indicate more anxiety or depression symptoms in the past week. The Dutch version of the HADS showed satisfactory validity and reliability [26]. In this study, the internal consistency (Cronbach’s alpha) of the anxiety scale was 0.83 and of the depression scale 0.75.

#### Disease characteristics

Due to privacy reasons, no information about the chronic conditions of the participants was provided by the benefits agency. This information was therefore derived through beneficiaries’ self reports. The questions concerning disease-characteristics were based on existing questionnaires [27] and recommendations of experts in the field. The following dichotomous disease-related variables were used: congenital disorder (yes/no), the nature of the disease process over time (“course of disease”: stable or positive vs negative or variable), daily use of medication (yes/no), need for medical devices in daily life, e.g. hearing aid and wheelchair (yes/no), tiredness (yes/no), limitations in use of fingers/hands, sight, hearing, and not being able to sit/stand for half an hour (yes/no), perceptible disability (yes/no).

### Statistical analysis

The Statistical Package for Social Sciences (SPSS) Windows version 16.0 was used for all the analyses. First, illness cognitions were analysed with descriptive statistics. Second, linear regression analyses were performed to predict the illness cognitions Acceptance, Helplessness and perceived Benefits by disease-characteristics while controlling for age and gender. Finally, linear regression analysis was performed to predict HRQoL, as expressed by the Mental and Physical Component Scale (RAND-36), Anxiety and Depression (HADS) by the illness cognitions, while controlling for disease-characteristics, age and gender. In line with Cohen [28], regression coefficients of binary-coded variables of 0.3 were considered small, 0.5 medium and 0.8 large. For continuous variables, regression coefficients of 0.1 were considered small, 0.3 medium and 0.5 large. A significance level of 0.05 was used for all analyses.

## Results

### Illness cognitions

The young adult beneficiaries reported the following mean item scores on the four-point ICQ subscales: Acceptance 2.95; Helplessness 2.10; perceived Benefits 2.86. In addition, to indicate the proportion of young adult beneficiaries who experienced at least some feelings of Acceptance, Helplessness and Benefits, the proportions of respondents with mean item scores ≥ 2 (indicating that the presence of the illness cognitions was “somewhat” to “completely”) were studied. It was found that 90.2% of the respondents experienced feelings of Acceptance, which was 83.8% in the case of perceived Benefits. 53.3% of the respondents had feelings of Helplessness to a greater or lesser degree.

Table 2 shows the standardized regression coefficients for the relation of disease- characteristics with Acceptance, Helplessness and Benefits (ICQ), corrected for age and gender. Those with a stable or positive course of illness reported more Acceptance (β = 0.20) and less Helplessness (β = −0.23) than those with a variable or negative course of disease. In addition, those who use medication reported less Acceptance (β = −0.15) and more Helplessness (β = 0.11), while using medical devices is associated with less Helplessness (β = −0.11). Furthermore, tiredness was associated with less Acceptance (β = −0.12) and more Helplessness (β = 0.13) and Benefits (β = 0.13); having problems with sitting was also associated with less Acceptance (β = −0.12) and more Helplessness (β = 0.17). Finally, respondents with a perceptible disability reported more Helplessness (β = 0.15). All significant regression coefficients are considered of small size.

**Table 2:** **Standardized regression coefficients β **^**1 **^**for the relation of disease characteristics with Illness cognitions (*ICQ *)**

	**Acceptance**	**Helplessness**	**Benefits**
	**β**	**β**	**β**
**Demographic characteristics**			
*Age*	0.01	0.02	0.05
*Female gender*^*a*^	0.07	−0.16***	0.14**
**Disease characteristics**			
*Congenital disorder*^*a*^	0.08	−0.07	−0.05
*Stable or positive course of illness*^*a*^	0.20***	−0.23***	0.05
*Medication*^*a*^	−0.15**	0.11*	−0.01
*Medical devices*^*a*^	0.11	−0.11*	0.00
*Tiredness*^*a*^	−0.12*	0.13*	0.13*
*Hands and/or fingers*^*b*^	0.03	−0.04	−0.04
*Vision*^*b*^	0.03	0.02	0.00
*Audition*^*b*^	−0.03	0.06	−0.03
*Sitting*^*b*^	−0.12*	0.17***	0.00
*Standing*^*b*^	0.07	0.06	0.08
*Disability is perceptible*^*a*^	0.03	0.15**	0.02
*F*	4.95	6.92	1.77
*df*	13,36	13,36	13,36
*R*^*2*^	0.15***	0.17***	0.06*

^1^Regression coefficients were based on linear regression analyses corrected for age and gender.

^a^coding: yes = 1, no = 0.

^b^coding: ‘problems with (the use of) …’ yes = 1, no = 0.

*p < 0.05; **p <0.01; ***p <0.001.

### Illness cognition in relation to health related quality of life, anxiety and depression

The contribution of illness cognition to Health Related Quality of Life, Anxiety and Depression, independent of the contributions of disease-characteristics, age and gender, are presented in Table 3. Feelings of Acceptance of the disease or disability were associated with better mental and physical HRQoL (β = 0.31, β = 0.16), less anxiety (β = −0.27) and less depression (β = −0.29), while having feelings of helplessness were associated with worse mental and physical HRQoL (β = −0.32, β = −0.15) and higher levels of anxiety (β = 0.28) and depression (β = 0.31). In addition, perceiving benefits of the disease or disability was associated with less depression (β = −0.14). The regression coefficients were small to medium.

**Table 3:** **Standardized regression coefficients β **^**1 **^**for the relation of Illness cognitions (*ICQ*) with HRQoL (*RAND-36*) **^**2**^**, anxiety and depression (*HADS*)**

	**MCS**	**PCS**	**Anxiety**	**Depression**
	**β**	**β**	**β**	**β**
**Demographic characteristics**				
*Age*	−0.02	−0.03	0.01	−0.04
*Female gender*^*a*^	−0.05	−0.07	0.08	−0.05
**Disease characteristics**				
*Congenital disorder*^*a*^	−0.06	0.07	0.03	0.05
*Stable or positive course of the disease*^*a*^	0.14*	0.21**	−0.02	−0.04
*Medication*^*a*^	0.03	−0.12**	−0.03	−0.01
*Medical devices*^*a*^	0.10	−0.13**	−0.06	−0.15*
*Tiredness*^*a*^	−0.17**	−0.14**	0.12	0.00
*Hands and/or fingers*^*b*^	0.13**	−0.10*	−0.10	−0.07
*Vision*^*b*^	−0.03	0.09*	0.01	−0.01
*Audition*^*b*^	0.01	0.00	−0.01	0.00
*Sitting*^*b*^	−0.01	−0.06	0.01	−0.02
*Standing*^*b*^	0.04	−0.36**	0.05	0.08
*Disability is perceptible*^*a*^	0.01	−0.06	−0.05	0.04
**Illness cognitions**				
*Acceptance*	0.31**	0.16**	−0.27**	−0.29**
*Helplessness*	−0.32**	−0.15**	0.28**	0.31**
*Benefits*	0.00	−0.05	−0.06	−0.14*
*F*	19.46	36.80	10.56	14.51
*df*	16,36	16,36	16,36	16,36
*R*^*2*^	0.46**	0.62**	0.32**	0.39**

^1^Regression coefficients were based on linear regression analyses corrected for disease characteristics, age and gender.

^2^Mental Component Scale (MCS) and Physical Component Scale (PCS).

^a^coding: yes = 1, no = 0.

^b^coding: ‘problems with (the use of) …’ yes = 1, no = 0.

*p <0.01; **p <0.001.

## Discussion

The present study focussed on generic illness cognitions of young adults with a disability benefit because of a somatic condition and on the relation of illness cognitions with emotional well-being in order to get insight in possible determinants of long-term adjustment in society. As far as we know, this is the first study on generic illness cognitions of young adults with a childhood-onset somatic condition. The results show that illness cognitions of these young adults are associated with emotional well-being.

A rather high level of acceptance and perceived benefits was found, indicating that the majority of the young adults with Wajong benefits in this study have learned to live with their illness or disability and even perceives some long-term beneficially consequences of their long term conditions. As they have been limited since childhood this may have influenced their acceptance and perception of possible benefits in a positive way. However, one should realize that the high levels of acceptance and perceived benefits we found in our study could be a result of selection bias. It is conceivable that young adults who have not accepted their disease yet, were less declined to participate in studies on psychosocial functioning, such as the present study. Helplessness in contrast, a cognition with a substantial negative impact, was present in a considerable part (53.3%) of the respondents. This indicates that the young adult beneficiaries in this study feel an inability to control a particular situation and emphasizes the negative aspects of their condition in daily functioning, which can lead to deterioration of their physical and psychological functioning [16,29]. Several disease-characteristics were associated with the illness cognitions, particularly those with a positive/stable course of illness scored higher on acceptance and lower on helplessness. However, those associations were not strong.

The illness cognitions acceptance and helplessness seemed to be associated with HRQoL, expressed by overall physical and mental quality of life, as well as with feelings of anxiety and depression in young adults with a disability benefit because of a chronic somatic condition. Though the direction of the correlation could not be established, it is plausible to assume that acceptance of the illness or disability, learning to live with it, influences HRQoL positively and reduces feelings of anxiety and depression. However, depression and anxiety can affect cognitions as well. Stronger feelings of helplessness appeared to be associated with worse HRQoL and higher levels of anxiety and depression. Young adult beneficiaries experiencing a great deal of helplessness are more likely to see their futures in adulthood as uncertain which could be a risk factor for the development of psychological distress over time. In addition, acceptance and helplessness were twice as strongly associated with mental quality of life than with physical quality of life, indicating that these illness cognitions are more important predictors of mental aspects of quality of life than of physical aspects. Levels of perceived benefits were not associated with levels of HRQoL and to a low degree to anxiety and depression. Our results suggest that acceptance and helplessness are possibly better predictors of distress than the perceived benefits of the chronic illness or disability. This is in line with research concerning the Benefit and Burden Scale for Children, a questionnaire that intends to measure potential benefit and burden of illness in children. It was found that disease-related burden was strongly associated with almost all psychological outcomes, while benefit finding was not [30,31].

The correlation between the illness cognitions and emotional well-being indicates that HRQoL, anxiety and depression hold disease-related elements. In other words, the illness cognitions of young adult beneficiaries are relevant for their psychological functioning. These findings are in line with studies in adult populations with chronic illness [32-34]. The modest contribution of disease-characteristics on HRQoL, anxiety and depression, supports the notion that there is only a weak relationship between biomedical parameters and psychosocial well-being. Illness cognitions on the other hand seem to contribute to individual differences in young adult beneficiaries regarding their psychosocial well-being and possibly long-term adjustment.

There are a number of shortcomings of this study that need to be addressed. First, the representativeness of the sample should be taken into account. The act Wajong is a Dutch benefit. Most countries have no specific benefit for young disabled people [35]. Furthermore, it is unknown which part of all young adults with a chronic illness or disability in the Netherlands apply for Wajong benefits. Therefore, it is advisable to be cautious and conservative while interpreting results of this study and extrapolating the findings to a larger population or to other countries. Another limitation is the response rate of 20%, though it is important to notice that this is a very average response rate among young adults with Wajong benefits [36,37]. Due to the growing interest in the labour market position of young adults claiming disability benefits, they receive too many invitations to participate in all the different studies. Moreover, it is likely that respondents did not fill in the questionnaire because the invitation letter was sent by the benefits agency. Although the questionnaire was anonymous, beneficiaries might be afraid of losing their benefit. Alternatively, those with better HRQoL were less eager to participate because of reluctance to feel stigmatized. On the contrary, among those who did participate social desirability could be a threat to the validity of the results in this study. Unfortunately, as a result of the need to respect the privacy of the beneficiaries, there is too little information regarding the non-responders to be able to pronounce upon selection bias. Anyway, it is hard to derive conclusions that are generally applicable to young adults with disability benefits because of the heterogeneity of both the illness diagnoses and illness severity in the Wajong population.

Another limitation concerns the assessment of illness cognitions. Though the ICQ was originally developed for people with a chronic illness, we used the ICQ also for young adults with a physical disability not caused by an illness. It is not known whether the ICQ is suitable for the assessment of illness cognitions in those with a pure physical disability but it can be assumed to be so. Furthermore, we did not study psychosocial factors that may predispose to individual difficulties in illness cognitions, for example personality. In future research this should be addressed.

Caution is called for interpreting the results of our study because the regression coefficients reflect that rather small portions of emotional well-being are explained by illness cognitions. In addition, causality could not be proven because of the cross-sectional nature of the study. Prospective, longitudinal research should confirm the direction of the present findings in order to be able to develop cognitive behavioural programs directed at the limitation of unhelpful cognitions and the development of personal skills needed to cope with the extra challenge of growing up with a chronic illness or disability. From a theoretical point of view, in depth exploration of IC including possible moderating effects of age and gender, could be interesting and add to the understanding of adjustment of young adults grown up with chronic disease.

Notwithstanding the limitations of the study, the results add to the understanding of adjustment of young adults with a childhood-onset chronic illness or disability. It is of importance that paediatric as well as adult healthcare providers and other professionals are aware of those psychosocial factors such as illness cognitions that contribute to emotional well-being of children, adolescents and young adults with somatic conditions. Special attention should be paid to coping with illness or disability during developmental transitions. For adolescents, transition into adulthood is a critical phase, characterised by multiple transitions including transition from family life to independent living and from education to employment. Because success in adulthood is closely related to positive social and emotional development earlier [38], it is recommended to monitor and support children and adolescents in an early stage. Early support and attention to social determinants of health would provide a chance to recruit patients with significant risks for unfavourable outcomes in later life and could stimulate patients in taking an active stance towards their medical condition. But also in adult healthcare, attention to possible consequences of illness cognitions and its relation to well-being seems sensible from a comprehensive, lifespan perspective on health care for young people with chronic conditions [39]. Special attention should be paid to the transfer from school to workforce participation. After transitioning from a school setting, an important metric for social success in adult life is employment [13]. There is increasing evidence that illness cognitions could play a role in work participation. Negative illness cognitions may lead to a feeling of being unwell that is not consistent with the diagnosed health condition, but equally may lead to incapacity to work. A review study of Hoving et al. [15] exploring the relationship between illness perceptions and work participation in patients with somatic diseases and complaints found promising evidence. The number of studies in the review was limited, but all included studies found significant associations between one or more illness perception dimensions and measures of work participation. In particular, all studies found that non-working people perceive more negative consequences of their illness. Although cause and consequence cannot be distinguished, these studies provided valuable information about illness perceptions in relation to employment.

## Conclusions

Although the study design does not justify conclusions with regard to causality, the results contribute to our understanding of the influence of young adult’s IC on their emotional well-being. Health care workers should pay systematic attention to the emotional well-being of patients growing up with a somatic condition and its relation with IC. Attention in an early stage of treatment yield opportunities for optimising the patient’s well-being and, because of this, maybe also their adaptation to society and a more fulfilling life. This support must be incorporated in a lifespan perspective in paediatric, transitional, and adult health care services for persons with a childhood-onset condition. A next step in research should be longitudinal studies focused on identifying individuals most likely to develop difficulties as a result of their IC, in order to be able to develop strategies for psychosocial support. Moreover, longitudinal research is needed on the relation between IC and successful labour participation in young adults who have grown up with a chronic somatic condition. It is also highly relevant to compare in future research different conceptualizations of illness cognitions, in addition to factors, such as coping strategies and social support, for the prediction of the health status in this specific patient group.

With the recognition of the psychosocial impact of a somatic condition in childhood on psychological well-being in later life comes the growing awareness of the need to develop psychosocial supportive programs. Systematic assessment of emotional functioning is not yet part of standard practice but effective routine assessment of emotional well-being could easily be implemented in daily clinical practice for children and adolescents through computerized and web-based patient reported outcomes [40-42]. The assessment of generic IC might be a valuable complementary tool for screening of psychological risk factors and tracing patients who may benefit from psychological interventions.

## Abbreviations

IC: Illness cognitions; HRQoL: Health related quality of life.

## Competing interests

The authors declare that they have no competing interests.

## Authors’ contributions

EV contributed to the concept and design of the study, carried out the data acquisition, analysed and interpreted the data and drafted the manuscript. HMS contributed to the concept and design of the study, analysed and interpreted the data and drafted the manuscript. HH, AE and MG contributed to the concept and design of the study and the manuscript. All authors read and approved the final manuscript.

## Authors’ information

EV is a PhD student at Pediatric Psychosocial department of the Emma Children’s Hospital Academic Medical Center (AMC) Amsterdam. Her research examines a large cross-sectional study (EMWAjong) directed at psychosocial functioning of adolescents and young adults with disability benefits because of a chronic somatic illness or disability since childhood and at factors affecting their vocational success.

HMS is health scientist and postdoctoral researcher within the Pediatric Psychosocial department of the Emma Children’s Hospital Academic Medical Center (AMC) Amsterdam who provides methodological support

HH is a professor in paediatrics and former chairman of the board of Emma Children’s Hospital Academic Medical Center (AMC) Amsterdam. He is now Chairman of the Global Health Initiative, Academic Medical Centre, University of Amsterdam.

AE is a professor of Health Psychology at the Leiden University and chair of the Health, Medical and Neuropsychology section. She specialises in the psychobiology of somatic disorders and symptoms. One of her aims is to understand the interaction between physical and psychological processes in healthy populations and somatic conditions, such as rheumatism or psoriasis and to develop innovative treatments based on that knowledge.

MG is a professor in paediatric psychology and head research of the paediatric psychology program in the Emma Children's Hospital AMC which is directed at three principal areas: studying the effects of a chronic disease or life-threatening disease on quality of life of children and young adults and family members; finding factors which predict these outcomes and development, implementation and evaluation of intervention programs. The department has extensive research experience in coordinating randomised controlled trials of psychosocial cognitive behavioural interventions with children with chronic diseases and cancer, and developing web-based interventions for young cancer survivors and their parents.
